# Tau-typing: a Nextflow pipeline for finding the best phylogenetic markers in the genome for molecular typing of microbial species

**DOI:** 10.1093/bioinformatics/btad425

**Published:** 2023-07-07

**Authors:** Matthew H Seabolt, Arun K Boddapati, Joshua J Forstedt, Konstantinos T Konstantinidis

**Affiliations:** Leidos Inc., Reston, VA 20190, United States; School of Biological Sciences, Georgia Institute of Technology, Atlanta, GA 30332, United States; Leidos Inc., Reston, VA 20190, United States; Leidos Inc., Reston, VA 20190, United States; School of Biological Sciences, Georgia Institute of Technology, Atlanta, GA 30332, United States; School of Civil and Environmental Engineering, Georgia Institute of Technology, Atlanta, GA 30332, United States

## Abstract

**Summary:**

Tau-typing is an integrated analysis pipeline for identifying genes or genomic segments whose phylogenetic resolving power most closely resembles the genome-wide resolving power of an input collection of genomes using the Kendall Tau rank correlation statistic. The pipeline is implemented in Nextflow and uses Docker and Singularity containers to ensure reliable scalability and reproducibility of results. This pipeline is particularly suitable for organisms for which whole-genome sequencing remains unaffordable or unscalable for routine applications, such as protozoan parasites which are not amenable to laboratory culture-based methods.

**Availability and implementation:**

Tau-typing is freely available at https://github.com/hseabolt/tautyping. The pipeline is implemented in Nextflow with Singularity support.

## 1 Introduction

Microbial genomes resistant or unamenable to laboratory cultivation, such as obligate protozoan parasites, prokaryotic pathogens like *Mycoplasma*, and many viruses, are among the most challenging to study from a public health perspective: they are evolutionarily divergent from most well-studied model genomes and their taxonomy often remains mired in uncertainty due to low-resolution, traditional methods employed (Waites and Talkington 2004, Letko *et al.* 2020, Keeling and Burki 2019, Dougan *et al.* 2022). Only a few high-quality genomes are available as reference data for such organisms due to difficulty in obtaining suitable quantities of template DNA for whole-genome sequencing (WGS). Thus, an open problem for public health initiatives studying these organisms is to address the scarcity of available genomic data in a scalable, affordable manner (Armstrong *et al.* 2019, Morris *et al.* 2019, Seabolt *et al.* 2021). This lack of data broadly limits testing many hypotheses regarding genome relatedness and evolution of important traits like differential virulence or the emergence of antimicrobial resistance. In addition, the frequency of recombination events between genomes may also introduce “noise” that can obscure evolutionary relationships. These challenges are particularly striking at the intra-species (e.g. strain, genotype, subtype) level, where the inability to differentiate strains without the resolution provided by WGS prevents outbreaks from being detected and linked to specific suspected sources (Tibayrenc and Ayala 2014). Thus, there is a great need for molecular typing techniques that can bring together the high resolving power afforded by WGS and the scalability of conventional PCR-based methods that are ubiquitous amongst molecular-enabled public health labs.

Here, we present Tau-typing, a fully automated, end-to-end Nextflow pipeline designed to leverage limited sets of WGS data to identify sets of high-resolution genomic markers or loci useful for conventional molecular typing (e.g. PCR assays) and/or *in silico* multilocus sequence typing (MLST) that their relatedness values best correlate with whole-genome-based relatedness of the same genomes, using the Kendall’s tau rank correlation statistic. Tau-typing has been packaged for deployment in diverse compute environments to enable availability to a wide audience of bioinformaticians as well as non-computational scientists and public health labs.

## 2 Pipeline and implementation

**Figure 1.**
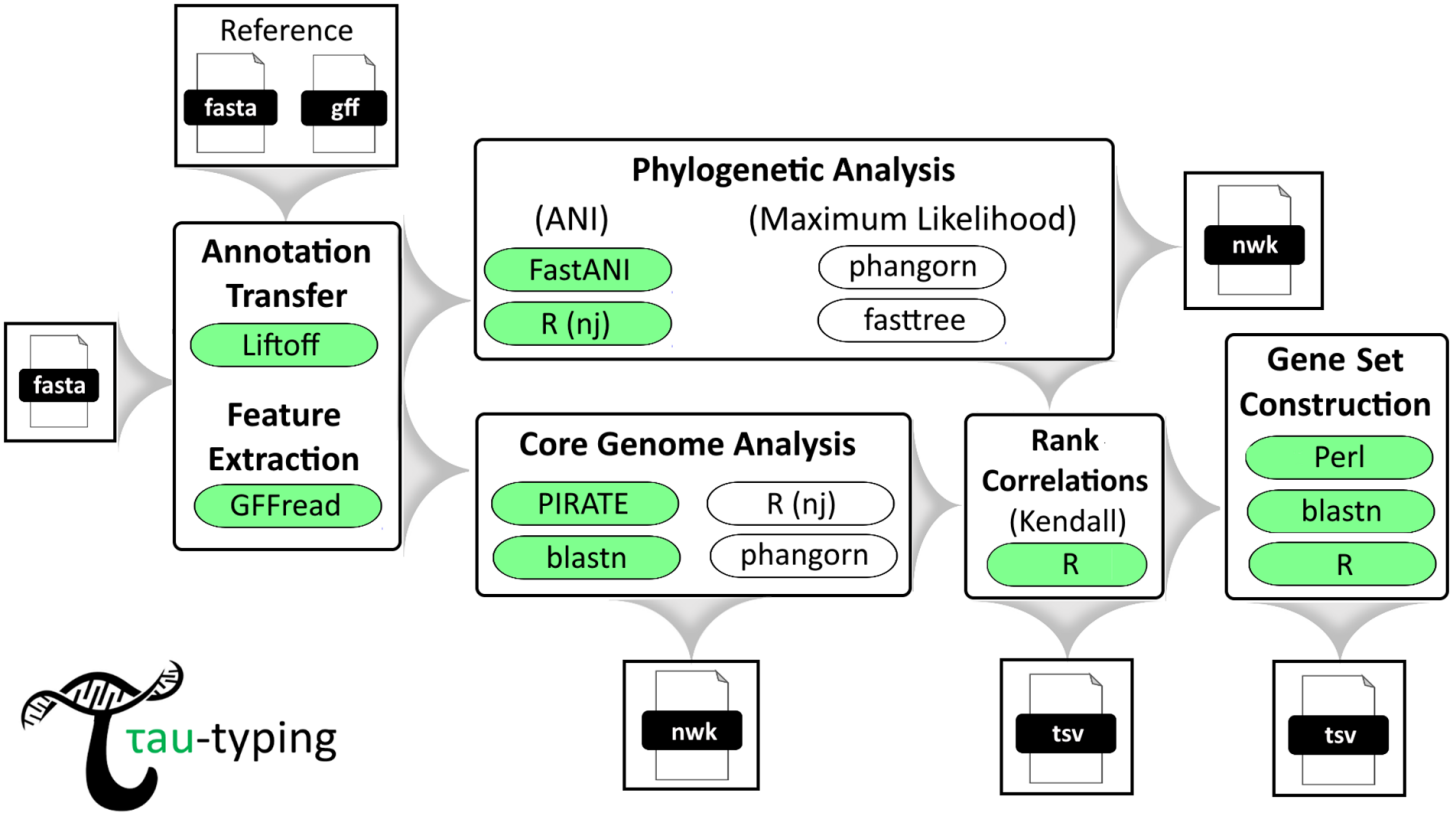
Tau-typing workflow diagram. The default analysis track (highlighted in green shaded ellipses) uses average nucleotide identity (ANI) and Kendall’s tau rank correlation to compute the best phylogenetic markers. An alternative analysis track using maximum likelihood and/or Spearman’s rho or Pearson’s *r*^2^ correlations are also implemented for users to choose from.

The Tau-typing pipeline computes a core genome from a set of input reference genomes, extracting and aligning genomic regions like protein-coding genes (Bayliss *et al.* 2019, Pertea and Pertea, 2020, Shumate and Salzberg 2021), and utilizes the Kendall tau rank correlation to directly compare the strength of correlation between the phylogenetic signal of each individual locus against the whole-genome-based signal to determine the best loci with this respect. Distance matrices are calculated from aligned sequences of each core gene according to the user’s specified distance metric, either nucleotide sequence identity computed using Blastn (Camacho *et al.* 2009) or maximum likelihood distances calculated using the R package phangorn (Schliep 2011). Similarly, the whole-genome-based matrix that the gene matrices are compared against using the tau correlation can be either the ANI (genome-aggregate average nucleotide identity) or maximum likelihood (ML) distances (Price *et al.* 2010, Jain *et al.* 2018); the latter based on the concatenated alignment of all core (shared) genes. Individual loci with the strongest correlation are then concatenated into super-alignments and their strength of correlation re-evaluated against the whole-genome to identify complementary groups of genes that boost resolving power when considered together. In this manner, our pipeline enables users to identify novel PCR targets and MLST schemas on-demand and agnostic of previously conceived typing schemes using only small fractions of the genome. Figure 1 illustrates the workflow in detail.

The main outputs are a summary MultiQC report (Ewels *et al.* 2016) and sorted lists of gene features and their associated tau correlation values. Users can focus on the genes with the highest correlation values for choosing the best markers, after taking into account any additional important factors such as ease of PCR amplification, which could be given higher priority over minor differences in correlation values (e.g. <0.05 difference in tau correlation). Additional details regarding specific tools, processing steps, output files, and options available to the user are described in Supplementary Materials and Methods. Note also that the ANI and ML methods implemented in this pipeline currently are not sensitive to detecting or correcting for recombination between the genomes being analyzed, which may result in discordant relationships between individual gene trees. If there is need to correct for the effect of recombination, the user could provide the corrected distance matrix (or a custom reference phylogenetic tree) instead of the default ANI/ML matrices to the pipeline. However, quantifying the magnitude of recombination, and the effects of (positive) selection on this magnitude, is generally a challenging task, and thus the non-corrected (for recombination) default distance matrices may be more realistic for identifying markers that work well even in light of substantial recombination. Accordingly, corrected matrices (for recombination) are generally not recommended.

Tau-typing is constructed using the Nextflow workflow management engine (Di Tommaso *et al.* 2017) following the nf-core style to maximize scalability, reproducibility of results, and maintenance of the codebase in the future (Grüning *et al.* 2018, Ewels *et al.* 2020, Bai *et al.* 2021). Each processing and/or analysis step of the pipeline is implemented as a module, enabling flexible use of executable code written in Bash, Python, Perl, or R scripts. Each module in the pipeline has additionally been containerized using software containers (Kurtzer *et al.* 2017) to facilitate full reproducibility and portability across computational environments such as different compute clusters or public cloud platforms (Microsoft Azure, Google Cloud Platform, Amazon Web Services). Nextflow provides out-of-the-box support for additional container technologies such as Docker, Podman, or Conda, as well as numerous batch schedulers to enable massive parallelization. To run the pipeline, the user need only have Nextflow and either Singularity, Docker, or Conda installed and available in the user’s PATH. Users provide input to the pipeline via a csv-formatted sample sheet containing two columns, a unique sample ID and a path to a FASTA assembly, plus a mandatory header line (“sample, fasta”). The pipeline can then be executed using the following command:

$ nextflow run hseabolt/tautyping -r 1.1 \

-profile singularity \

––input <SAMPLESHEET> \

––outdir <OUTPUT>

## 3 Application

While our primary envisioned use cases are microbial eukaryotes, we tested three datasets to determine the robustness of the pipeline’s output across a breadth of taxa: (i) 20 viral genomes of SARS-CoV-2 variants obtained from GISAID (Elbe and Buckland-Merrett 2017) and NCBI, (ii) a bacterial dataset of 12 representative *Escherichia coli* closed genomes used previously (Konstantinidis *et al.* 2006), and (iii) a microbial eukaryote set of 35 *Giardia duodenalis* assemblage B (protozoan) genomes (Tsui *et al.* 2018). Each of these cases draws a limited sample of genomes from the same species, capturing unique biological considerations such as mutation rate, genetic distance differentiating strains, and overall quality of genome assemblies. Each dataset was benchmarked used the pipeline’s default analysis track (ANI/sequence identity + Kendall’s tau rank correlation) and version 1.1 of the pipeline’s code. Each run was conducted on an HPC cluster using the Grid Engine batch scheduler. Runtimes for each dataset were generally proportional to the size of the input genomes in terms of nucleotide base pairs, ranging from 2m34s (SARS-CoV-2) up to 5h32m for the largest dataset (*G.duodenalis*). Benchmark results are provided in Supplementary Table S1. Reference genomes and their respective annotation files are provided with the source code in the “assets/reference” folder. Test data are included in the “data” folder for reproducibility, except for SARS-CoV-2 genomes downloaded from GISAID.

Data files from multiple-sequence alignments of each gene and gene set analyzed, plus the respective distance matrix and phylogenetic tree, are included in the pipeline’s output to enable users to easily conduct further analysis, such as identifying primer binding sites, cluster sequences, etc. By analyzing groups of genomes showing different levels of divergence, e.g. more clonal (SARS-CoV-2) versus higher intra-group sequence diversity (*E.coli* or *Giardia*), we show that this pipeline is most suitable for bacterial and microbial eukaryote genomes that are related in the range of 95–99.9% ANI (Supplementary Fig. S1). Among the top 10 performing genes for *E.coli* and *G.duodenalis*, all concatenated gene sets of two genes or more returned correlation coefficients to whole-genome between 0.801–0.889 and 0.601–0.730, respectively, while the range of comparable SARS-CoV-2 sets was −0.061–0.362. This result is likely driven by the small number of genes in the SARS-CoV-2 genome compared to *E.coli* and *Giardia*, where the top 10 genes exhibit mutation rates proportional to the genome-wide rate, while the top-ranking genes from SARS-CoV-2 are poorly comparable with genome-wide rates of evolution, likely due to lack of evolutionary time to accumulate enough fixed mutations or the high rate of recombination observed in this virus (Turakhia *et al.* 2022). A further example of Tau-typing’s practical application is shown in Supplementary Fig. S2, demonstrating that highly similar phylogenetic resolving power to genome-based phylogeny/ANI can be achieved using very few genetic markers (*n* = 3–10), clearly surpassing the resolving power of the 8-gene PubMed MLST scheme for *E.coli* (Jolley and Maiden 2010; Github: https://github.com/tseemann/mlst). Two prior studies using a precursor (non-standardized, manual) method to the one described here for identifying suitable MLST loci confirmed that as few as three genes can provide robust resolution for accurate genomotyping (Konstantinidis *et al.* 2006, Seabolt *et al.* 2021). To maximize practical value in their own use cases, users of our pipeline can limit the number of top performing genes to use for constructing concatenated gene sets (for example, to use only the three best performing genes) or can leave the default of 10 genes in place and filter/select best-performing results using additional selection criteria such as the availability of good primer binding sites and/or free of spatial autocorrelation.

## 4 Conclusion

In the present work, we present an eponymous end-to-end Nextflow pipeline, Tau-typing, tuned to identify sets of high-resolution genomic loci from limited sets of whole-genome sequences. Using the Tau-typing pipeline, users with minimal bioinformatics or programming backgrounds can quickly and easily generate sets of loci that are suitable for high-resolution typing, enabling improvements in routine comparative genomics, public health surveillance activities, rapid response to emerging outbreaks, and more. Extensions to the core use cases of Tau-typing, such as the use of a custom phylogenetic tree to specify relationships between lineages (e.g. for biogeographic or host-association analyses) are implemented to further complement the value of this pipeline to users interested in identifying genes showing signal of biogeographic endemicity or host-specific adaptations. Sensitivity to recombination remains a challenge for Tau-typing’s core algorithm. Therefore, in use cases that is viewed necessary to correct for the effects of recombination, we suggest users to provide a custom phylogenetic tree constructed using coalescent methods or which has had branch lengths corrected for recombination. We expect results produced by our pipeline to be most impactful for taxa such as protozoa which remain reliant on morphology or conserved loci for primary typing/taxonomic assignment by introducing high-resolution markers that can easily be compared across studies over time. We welcome collaborative improvements and additional suggestions for extensions to the pipeline as new use cases are envisioned and benchmarked.

## Supplementary Material

btad425_Supplementary_Data

## Data Availability

The Tau-typing code is available on Github at https://github.com/hseabolt/tautyping and released under the MIT license. NCBI and GISAID accession numbers for each genome used in benchmarking datasets are provided in Supplementary Table S2. Test data obtained from NCBI and used for benchmarking are provided alongside the Tau-typing source code in Github.
