# Supplementary material for: Tau-typing: a Nextflow pipeline for finding the best phylogenetic markers in the genome for molecular typing of microbial species
Source: Bioinformatics. 2023 Jul 7;39(7):btad425. doi: 10.1093/bioinformatics/btad425 (PMC10336021; doi:10.1093/bioinformatics/btad425)
Supplement: btad425_Supplementary_Data [file btad425_supplementary_data.pdf]

Supplementary Materials and Methods

Pipeline Input

The Tau-typing pipeline accepts a csv (comma-delimited) sample sheet containing a header line and two corresponding columns: a unique sample ID, followed by an accessible file path to a FASTA genome assembly. The header line is required and the column names "sample,fasta" are strictly enforced by the pipeline's code. Genome assemblies should be suitably representative of the known genetic diversity of the organism of interest, to the extent possible by available data. We recommend users to compare genomes at the species level (~90-100% ANI) -OR- at the intra-species (=clonal complex, strain, genotype, subtype, etc.) level (>= 99% ANI) separately to capture the best phylogenetic markers at different granularities of relatedness. Likewise, if most genomes being compared are very closely related (~99-100% ANI), we suggest users not include more distant genomes. Paths to FASTA assemblies can be on local disk or point to an accessible location such as an S3 bucket hosted on Amazon Web Service (AWS) and can optionally be gzip-compressed. Sample IDs should be simple, unique names and may contain underscores or hyphens. These IDs will be used as prefixes by the pipeline when auto-generating output results files, so they should be free of spaces or other special characters that may disrupt reading/writing of files by the user's operating system.

The pipeline also requires a FASTA assembly and GFF annotation file from a closely related genome to the genome assemblies given in the sample sheet. The user optionally may also specify a text file containing feature types of interest, e.g. "gene", "CDS", etc., using the '--feature_types' parameter. An example sample sheet and feature type file are included with the pipeline source code in the 'assets' directory for reference.

Preprocessing and annotation transfer

Pre-processing consists of simple checks to confirm that the reference genome and its annotations conform to the format expected by Liftoff (Shumate and Salzberg, 2021). Once this is cleared, the reference FASTA, GFF, and any feature type specifications are passed to Liftoff along with all the remaining genomes in the sample sheet. Briefly, Liftoff extracts the reference features and maps them to the target genomes using Liftoff's default alignment parameters of 50% of the query length aligned with >= 50% nucleotide identity to be considered as a homologous match. These parameters are suitable for the reference genome to originate from a related species if a high-quality assembly and annotations are not available from the target species, and the user is able to configure these to suit their needs if required. The output from Liftoff is a GFF file per target genome containing all successfully mapped reference annotations, plus a second text file containing a list of all features that were unable to be mapped to the target.

Computing a core genome of genes/genomic features

The set of transferred annotations (GFF files) is provided as input to the program PIRATE (Bayliss et al., 2019), which computes a core set of genes (i.e. those present in all genomes) from the total given input set. Using sequence alignments output from PIRATE, distance matrices are computed for each gene feature using Blastn or the phangorn package in R (Camacho et al. 2009, Schliep 2011). Distance matrices are output as tab-delimited files with the extension '.dist' and are additionally visualized using neighbor-joining and provided for the user's reference.

Evolutionary relationships using whole-genome sequences (WGS)

The 'gold standard' or 'ground truth' evolutionary relationships against which all other relationships based on individual gene features are compared can be obtained in one of three ways in this pipeline: (i) using the program FastANI (Jain et al., 2018) to compute pairwise ANI distances between all

genome pairs, the results of which are transformed into a distance matrix, (ii) by concatenating all core gene alignments into a super-alignment and using this to compute maximum likelihood distances between all sequences with phangorn, or lastly (iii) by allowing the user to provide a Newick-formatted phylogenetic tree in which the leaves correspond to the set of input genomes and which includes branch lengths. The former two options are agnostic towards any prior classification schemas, while the third option is provided to extend this pipeline's utility to users who may wish to evaluate gene features against a non-genetic or *ad hoc* set of relationships. For example, a phylogeny showing biogeographic relationships measured as earth-distances (in kilometers) could be evaluated to determine which genes show the strongest signal of biogeographic endemicity. A second example using host-origin relationships could similarly be evaluated to search for genes that are potentially involved in host-specific adaptations. A custom phylogenetic tree can be specified using '--custom_tree'. If this option is activated, the required distance matrix is obtained by computing pairwise cophenetic distances between all leaves on the given tree. By default, the ANI methodology is used in this pipeline but may be switched to maximum likelihood using the '--distance' parameter.

Comparing individual gene features against WGS

Strength of correlation between distance matrices from WGS and individual gene features is calculated using the cor.test() function in R. We provide users with the ability to specify whether they wish to use Kendall's tau rank correlation (default), Spearman's rho, or Pearson's product-moment correlation coefficient. We recommend using the Kendall tau method, as genetic distances very rarely meet the assumption of normality required for Pearson's method and are often not monotonic as assumed by the Spearman method. Briefly, the tau rank correlation is a non-parametric statistic that measures the strength of association (correlation) between two ordinal variables. Tau is computed by ranking the data in each variable and subsequently comparing the ordering of ranks to estimate correlation. Following calculation of correlations, an output file is produced and sorted in descending order by correlation coefficient. We visualize a distribution of correlation coefficients alongside the sorted table of correlations. Both files are ingested by MultiQC (Ewels et al., 2016) as part of the final output report in HTML format.

Evaluating concatenated sequences of the best-performing genes against WGS

It is often the case that individual genes are not sufficient to serve as a standalone marker capable of perfectly reflecting WGS relationships. Thus, it is advantageous to combine genes with high correlation with WGS into sets and re-evaluate the strength of correlation of the set vs WGS. Here, the user is given the option to control the parameters guiding set construction with the '--n' and '--k' arguments. We use a power set algorithm to generate non-redundant concatenated FASTA files of the highest ranked *n* genes (by default, 10). This algorithm will generate 2^n sets (-1 if we discard the empty set), thus for n=10, 1024 sets ranging from size *k*={1..10} are generated. Further control is given to the user with the parameters '--kmin' and '--kmax' to restrict the values of *k* if desired. Gene sets are provisioned with a unique numerical ID, FASTA files are concatenated to form a (pseudo) super-alignment, transformed into a distance matrix, and compared against the WGS distance matrix in the same manner described above for individual genes. Computed rank correlations for all sets are collated and sorted as a CSV-formatted output file for the user's review. A distribution of correlation coefficients is likewise produced as described previously. Both files are ingested by MultiQC as part of the final output HTML report.

References

1. Bayliss, S. C., Thorpe, H. A., Coyle, N. M., Sheppard, S. K., & Feil, E. J. (2019). PIRATE: A fast and scalable pangenomics toolbox for clustering diverged orthologues in bacteria. *Gigascience*, *8*(10), giz119.
2. Camacho C, Coulouris G, Avagyan V, Ma N, Papadopoulos J, Bealer K, Madden TL (2009). BLAST+: architecture and applications. *BMC Bioinformatics,* **10**, 421.
3. Ewels, P., Magnusson, M., Lundin, S., & Käller, M. (2016). MultiQC: summarize analysis results for multiple tools and samples in a single report. *Bioinformatics*, *32*(19), 3047-3048.
4. Jain, C., Rodriguez-R, L. M., Phillippy, A. M., Konstantinidis, K. T., & Aluru, S. (2018). High throughput ANI analysis of 90K prokaryotic genomes reveals clear species boundaries. *Nature communications*, *9*(1), 5114.
5. Jolley KA, Maiden MC. (2010) BIGSdb: scalable analysis of bacterial genome variation at the population level. *BMC Bioinformatics*. **11,** 1-11.
6. Schliep, K. P. (2011). phangorn: phylogenetic analysis in R. *Bioinformatics*, *27*(4), 592-593.
7. Shumate, A., & Salzberg, S. L. (2021). Liftoff: accurate mapping of gene annotations. *Bioinformatics*, *37*(12), 1639-1643.

Supplementary Tables

Table S1: Benchmarking of 3 datasets from SARS-CoV-2 (viral), *E. coli* (Prokaryote), and *Giardia duodenalis* (Microbial Eukaryote).

	SARS-CoV-2	*E. coli*	*G. duodenalis*
**Reference Genome**	NC_045512	CFT073	GS
**Reference NCBI Acc.**	NC_045512.2	GCF_021559855.1	GCA_000498735.1
***n* Genomes**	20	12	35
**Genome Length (bp)**	29,903	5,236,926	12,009,633
***n* Core Genes**	7	2290	3157
***n* Computed Sets**	31	1023	1023
**Runtime**	2m 34s	2h 26m 29s	5h 32m 21s
**Max CPUs**	2	8	16
**Max RAM**	2 GB	16 GB	128 GB
**No. Jobs**	297	18,493	20,928

Table S2: Sample IDs and NCBI accession numbers for all benchmarking data used in pipeline testing

**Table S2: Benchmarking Accesions IDs**		
Sample ID	NCBI Accession	Dataset
Ecoli_CFT073	GCF_021559855.1	*E. coli*
Ecoli_E2348-69	GCF_014117345.2	
Ecoli_O42	GCA_014462355.1	
Sdysenteriae_SWHEFF49	GCF_022354085.1	
Ecoli_O157-EDL933	GCF_000732965.1	
Ecoli_O157_Sakai	GCF_000008865.2	

Ecoli_E24377A	GCF_000017745.1	
Ssonnei_53G	GCF_000283715.1	
Sflexneri_301	GCF_000006925.2	
Ssonnei_SE6-1	GCF_013374815.1	
Ecoli_HS	GCF_022453605.1	
Ecoli_K12	GCA_028644645.1	
BAH15c1	GCA_001543975.1	*Giardia duodenalis* assemblage B
GS	GCA_000498735.1	
GSMH7	GCA_000182405.1	
SRR3177748	SRR3177748	
SRR3177755	SRR3177755	
SRR3177763	SRR3177763	
SRR3177816	SRR3177816	
SRR3177826	SRR3177826	
SRR3177835	SRR3177835	
SRR3177841	SRR3177841	
SRR3177858	SRR3177858	
SRR3177862	SRR3177862	
SRR3177903	SRR3177903	
SRR3177917	SRR3177917	
SRR3177926	SRR3177926	
SRR3177928	SRR3177928	
SRR3177929	SRR3177929	
SRR3177930	SRR3177930	
SRR3177931	SRR3177931	
SRR3177933	SRR3177933	
SRR3177934	SRR3177934	
SRR3177936	SRR3177936	
SRR3177937	SRR3177937	
SRR3177947	SRR3177947	
SRR3177948	SRR3177948	
SRR3177952	SRR3177952	
SRR3177955	SRR3177955	
SRR3177962	SRR3177962	
SRR3177974	SRR3177974	
SRR3178008	SRR3178008	
SRR3178009	SRR3178009	
SRR3178011	SRR3178011	
SRR3178013	SRR3178013	
SRR3178014	SRR3178014	

SRR3178015	SRR3178015	
alpha	EPI_ISL_1214361	SARS-CoV-2
B.1.617.3	OU208276.1	
beta	EPI_ISL_745110	
delta	EPI_ISL_1718630	
ep_B.1.427	EPI_ISL_847569	
ep_B.1.429	EPI_ISL_847621	
eta	EPI_ISL_954180	
gamma	EPI_ISL_804824	
iota	EPI_ISL_794226	
kappa	EPI_ISL_2088240	
mu_B.1.621	OU351975.1	
NC_045512.2	NC_045512.2	
om_B.1.1.529	OP295756.1	
om_BA.1.1	OP295757.1	
om_BA.1	OX315743.1	
om_BA.2	OX315675.1	
om_BA.3	OW901148.1	
om_BA.4	OP093374.1	
om_BA.5	OP603965.1	
zeta	EPI_ISL_717936	

* Accession IDs for SARS-CoV-2 genomes beginning with 'EPI' refer to GISAID identifiers.

Supplementary Figures

Figure S1: Density distributions of Kendall tau rank correlations from all 3 data sets, separated as individual genes and computed sets of genes ranging from *k*={2..10}. Distributions were plotted in R using the base R plot() function.

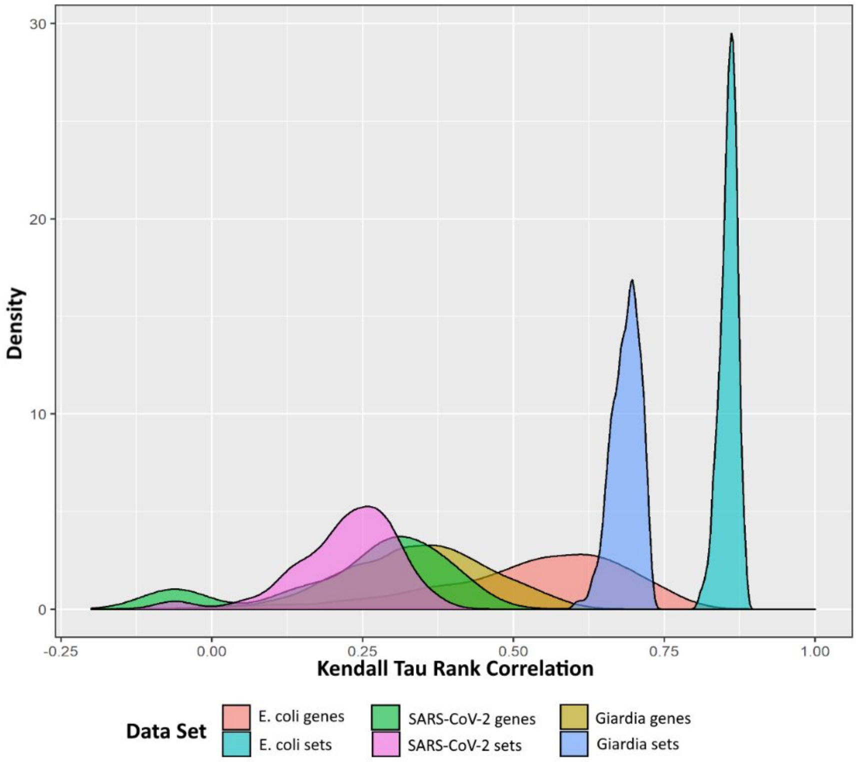

Figure S2: Example comparison of phylogenetic trees based on ANI/sequence identity of 12 *E. coli* group genomes. Trees are computed using neighbor-joining from ANI distance matrices. (a) Relationships based on whole genomes (approx. 5.27 Mbp), (b) the best-performing set of genes (n=8; tau=0.89, 7188 bp), and c) PubMed *E.coli* MLST scheme (n=8, 4098bp). Branches shown in red denote relationships that differ in (b) and (c) from those in (a).

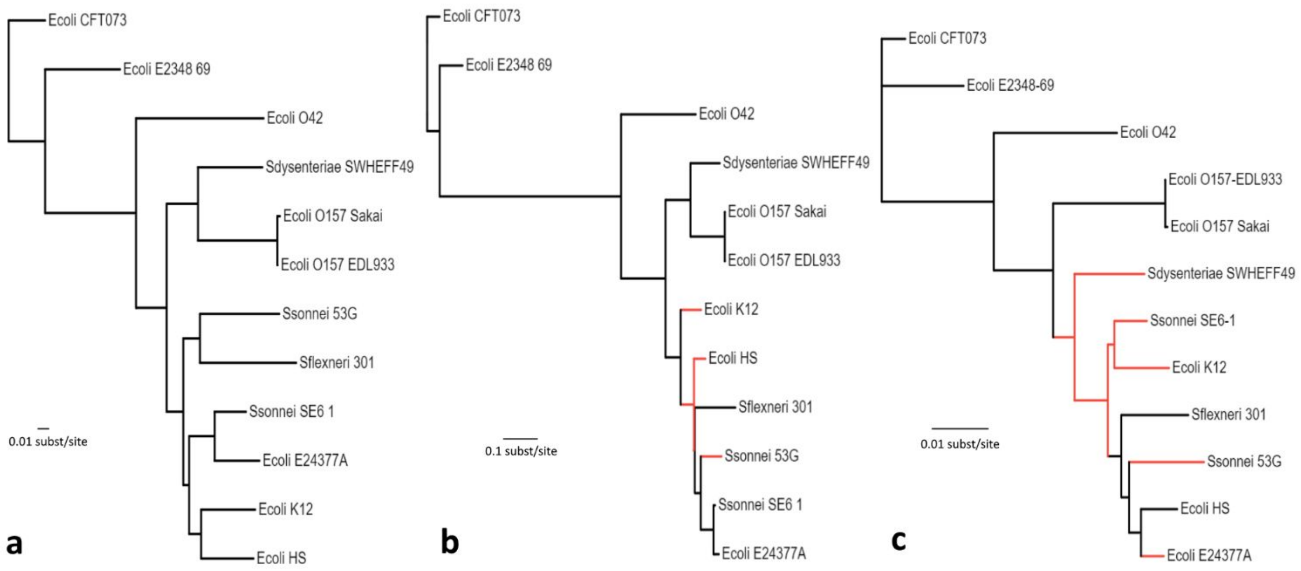
Ecoli CFT073
Ecoli E2348 69
Ecoli O42
Sdysenteriae SWHEFF49
Ecoli O157 Sakai
Ecoli O157 EDL933
Ssonnei 53G
Sflexneri 301
Ssonnei SE6 1
Ecoli E24377A
Ecoli K12
Ecoli HS
0.01 subst/site
a
Ecoli CFT073
Ecoli E2348 69
Ecoli O42
Sdysenteriae SWHEFF49
Ecoli O157 Sakai
Ecoli O157 EDL933
Ecoli K12
Ecoli HS
Sflexneri 301
Ssonnei 53G
Ssonnei SE6 1
Ecoli E24377A
0.1 subst/site
b
Ecoli CFT073
Ecoli E2348-69
Ecoli O42
Ecoli O157-EDL933
Ecoli O157 Sakai
Sdysenteriae SWHEFF49
Ssonnei SE6-1
Ecoli K12
Sflexneri 301
Ssonnei 53G
Ecoli HS
Ecoli E24377A
0.01 subst/site
c
